# Does type of active workstation matter? A randomized comparison of cognitive and typing performance between rest, cycling, and treadmill active workstations

**DOI:** 10.1371/journal.pone.0237348

**Published:** 2020-08-07

**Authors:** Kayla M. Frodsham, Nicholas R. Randall, Kaylie A. Carbine, Rebekah E. Rodeback, James D. LeCheminant, Michael J. Larson

**Affiliations:** 1 Department of Psychology, Brigham Young University, Provo, UT, United States of America; 2 Department of Nutrition, Dietetics, and Food Science, Brigham Young University, Provo, UT, United States of America; 3 Neuroscience Center, Brigham Young University, Provo, UT, United States of America; University of Kent, UNITED KINGDOM

## Abstract

Active workstations are associated with improved health outcomes, but differences in cognitive and typing outcomes between the types of active workstations are unclear. We addressed two main questions: (1) Are there differences in cognitive and typing performance between seated and active workstations? (2) Are there differences in cognitive and typing performance between cycling and treadmill workstations, specifically? Participants included 137 healthy young adults (74 female, mean age = 20.8 years) who completed two sessions. At session one (baseline), all participants completed cognitive and typing tests including the Rey-Auditory Verbal Learning Test, Paced Auditory Serial Addition Test, a typing test, and a flanker task while sitting at rest. At session two, participants were randomized to an active workstation group (treadmill or cycling desk) during which they performed the tests listed above in a randomized fashion, using alternate versions when available. Participants showed significantly better attention and cognitive control scores during the active session as compared to the seated session, but worse verbal memory scores during the active session. Participants were faster and more accurate at typing during the active session relative to the seated session. There were no significant differences between cycling or treadmill workstations on any cognitive or typing outcomes. Improvements during active sessions may be influenced by practice effects, although alternate forms were used when possible. We conclude that active workstations do not seem to largely impact cognitive abilities, with the exception of a slight decrease in verbal memory performance. Findings suggest active workstations, whether walking or cycling, are useful to improve physical activity, particularly when completing tasks that do not require verbal memory recall.

## Introduction

On average, children and adults in the United States spend approximately 50% of their waking hours in a sedentary state [1]. Sedentary time is a risk factor for multiple health difficulties including obesity, diabetes, inappropriate weight gain, and cardiovascular disease [2]. Specifically, sedentary jobs, such as service-providing jobs (e.g., retail, finance, business, education), have increased steadily since the 1960’s [2, 3]. As such, the workplace is an opportune and practical place to intervene in order to decrease sedentary time and improve health outcomes. Active workstations provide an opportunity for increasing workplace-related physical activity. Yet, studies comparing the effects of different types of active workstations (e.g., standing desks, walking desks, elliptical trainers, cycling desks) on cognitive function and work performance are limited [4, 5].

Active workstation use is associated with increases in physical activity. A systematic review concluded that treadmill desks in work settings decrease sedentary time; on average participants took more than 2,000 steps per day when a treadmill desk was available than when seated [5]. Active workstations are also associated with an increase in energy expenditure relative to sitting, with a large effect size [i.e., Cohen’s *d* effect size ranging from .83 to 16.75 [6]. Calorie expenditure increases from sitting (ranging from 72 kcal to 88 kcal) to active (ranging from 191 kcal to 376 kcal) workstations [6]. In a meta-analysis, cycling workstations showed a greater effect on energy expenditure compared to treadmill workstations [cycling standard mean difference = 6.50, treadmill standard mean difference = 2.23 [7].

Active workstations are associated with several health benefits in addition to the increase in physical activity described above. Active workstation use is related to weight loss, fat loss, and smaller hip and waist circumference [5, 8, 9]. For example, one study showed a modest effect with weight changing from 86.3 kilograms (kg; *SD* = 26.5 kg) at baseline to 85.1 kg (*SD* = 25.6 kg) 12 months later [8]. Participants in the study increased from 70 minutes/day (*SD* = 25) of average physical activity at baseline to 128 minutes/day (*SD* = 62) at six months (63% of the increase due to desk use), and 109 minutes/day (*SD* = 62) at 12 months [90% of the increase due to desk use [8]. High responders lost 4 kg on average (*SD* = 4) after 12 months, suggesting there may be variability in weight loss among active workstation users [8]. Active workstations are also related to decreased total cholesterol, decreased blood glucose, decreased insulin levels, and lower blood pressure when compared to sedentary work time [5, 7, 10]. While there are clearly health benefits to active workstations, it is important to note some studies do not find significantly different health outcomes after active workstation interventions (e.g., systolic and diastolic blood pressure) and some studies suggest possible risks associated with the use of active workstations [e.g., musculoskeletal, leg/foot swelling] [7, 9].

Although the health benefits of active workstations such as treadmill or cycling desks are relatively clear, the effects of active workstations on cognitive and work performance are mixed. Most studies to date suggest no difference or small improvement in cognitive performance when comparing active workstations to control conditions. For example, multiple reviews suggest cognitive skills such as attention, processing speed, short-term memory, reasoning, and reading comprehension were similar when participants used an active workstation compared to a control condition [4–6, 10]. The prevailing evidence across studies similarly shows a pattern of no differences (or a few improvements) in cognitive performance (e.g., executive functioning, working memory, attention, academic functioning) when comparing people who used active workstations to people in various sedentary control conditions [11–21].

There are some exceptions where researchers have found detrimental effects of active workstations on cognitive performance compared to seated or resting conditions. For example, a few specific abilities measured in test batteries (e.g., planning, working memory, math) were lower during active workstation conditions compared to resting or seated control conditions [5, 13, 14]. One study found that performance on several cognitive abilities (e.g., attention/working memory, delayed verbal memory) were lower during an active workstation condition (treadmill) than during the control condition [22]. That said, as noted above, the majority of studies show few-to-no difficulties on cognitive performance and it seems health improvements from active workstation use outweigh any small declines on specific cognitive tasks [4–6, 10–21].

The literature is mixed on the effects of active workstation use on typing performance. The majority of reviews suggest either no effect or a detrimental effect of active workstation use on typing performance, both for cycling and treadmill desks [4–6, 10, 19]. Several primary studies find similar results: no effect [20, 23–25] or detrimental effect [16, 23, 24, 26] of active workstation on typing performance, sometimes with mixed results depending on the rate of walking/cycling used for the workstation. In summary, research to date show variability in the relationship between active workstations (cycling and treadmill) and typing performance, overall suggesting detrimental or insignificant effects.

While the literature on the benefits or decrements from active workstation use is growing, there are few studies that have directly compared cognitive and typing performance between different types of active workstations such as treadmill desks and cycling desks. The few papers that have assessed differences in cognitive performance between people using treadmill and cycling desks tend to employ within-subject designs with random assignment to order of conditions. One study found cognitive performance was better during treadmill and cycling conditions compared to a seated condition on all three domains assessed (psychomotor, working memory, executive functioning), but participants had decreased performance in psychomotor functioning on the treadmill compared to the cycling condition; there was no significant difference between cycling and treadmill on working memory or executive functioning [21]. In another study, most cognitive performance outcomes (reading comprehension, attention, perceptual performance, executive functioning) did not significantly differ between type of dynamic workstation (treadmill, cycling, elliptical) and sitting; the one exception was decreased accuracy on a working memory task for cycling at 40% heart rate reserve compared to the sitting condition [27]. Finally, objective web-based search task performance was lower during a cycling desk condition compared to a standing condition, but not significantly different between a treadmill condition and either stationary condition [standing or sitting] [12].

Regarding typing performance, one set of researchers found no significant difference between the cycling condition and the sitting condition, but significantly slower typing speeds for the treadmill condition compared to the sitting condition, suggesting impaired typing performance at a treadmill desk but not at a cycling desk [27]. Another group found both treadmill and cycling conditions had decreased typing outcomes compared to a sitting condition; further, the researchers concluded that treadmill had a larger negative impact on typing performance while cycling had a smaller negative impact on typing performance, although no direct comparison was reported in the paper [they instead used qualitative comparisons of effect sizes] [23]. Making inferences about the superiority of one workstation over another is difficult because many of the studies above do not report direct statistical comparisons between cycling and treadmill outcomes. Instead, many studies report statistical differences between each active workstation and a control condition. As such, direct comparisons of cognitive and typing performance between different types of active workstations is a significant gap in the existing literature that needs to be addressed.

To address the need for randomized studies directly comparing cognitive and typing performance between cycling and treadmill workstations, we used a seated baseline testing session followed by a randomly assigned active session (either a cycling desk or a treadmill desk). Our study was guided by the following questions and hypotheses: (1) Is there a difference in cognitive performance between seated and active workstations (collapsed across cycling and treadmill workstations)? Based on existing literature, we predicted no difference in cognitive outcomes between seated and active workstations. (2) Is there a difference in typing skills between seated and active workstations (collapsed across cycling and treadmill workstations)? We predicted typing scores would be significantly lower when using the active workstation compared to the seated workstation. (3) Is there a difference in cognitive performance between cycling and treadmill workstations? We predicted no difference in cognitive performance between cycling and treadmill workstations. (4) Is there a difference in typing skills between cycling and treadmill workstations? We predicted no difference in typing skills between cycling and treadmill workstations.

## Materials and method

### Participants

This project was pre-registered on the Open Science Framework (OSF) and the study data and study information are available at the following link: https://osf.io/f4ej3. All procedures were approved by the Brigham Young University Institutional Review Board; participants provided written, informed consent and study procedures followed the guidelines outlined in the Declaration of Helsinki. Healthy young adults (*n* = 137, 74 females, mean age = 20.82, *SD =* 1.89; mean weight = 72.48 kg, *SD* = 17.74 kg; mean body mass index (BMI) = 24.13 kg/m^2^, *SD* = 3.70 kg/m^2^) were recruited from undergraduate psychology courses. We chose to enroll at least 100 participants (with the end of a university semester being the final stopping point) based on small effects found with comparable sample sizes in previous studies [22, 26]. We initially pre-registered only 102 participants, but by completing data collection to the end of the academic semester ended with 137 total participants, a slight deviation from the pre-registration.

Exclusion criteria included impairments in mobility, psychiatric or neurologic diagnosis, learning disability, attention-deficit/hyperactivity disorder, or English as a second language. Using a random number generator, participants were randomized for their active workstation session to either the treadmill group (*n* = 73, 37 females, mean age = 20.79, *SD* = 2.07; mean weight = 72.83 kg, *SD* = 18.19 kg; mean BMI = 24.17 kg/m^2^, *SD* = 3.93 kg/m^2^) or the cycling group (*n* = 64, 37 females, mean age = 20.86, *SD* = 1.67; mean weight = 72.07 kg, *SD* = 17.34 kg; mean BMI = 24.08 kg/m^2^, *SD* = 3.45 kg/m^2^). There were no significant differences in age, *t*(135) = -0.20, *p* = .84, proportion male/female, *χ*^*2*^(1) = .70, *p* = .40, weight, *t*(132) = 0.25, *p* = .81, or BMI, *t*(132) = 0.13, *p* = .89, between the treadmill and cycling desk conditions.

### Procedures

**Fig 1:**
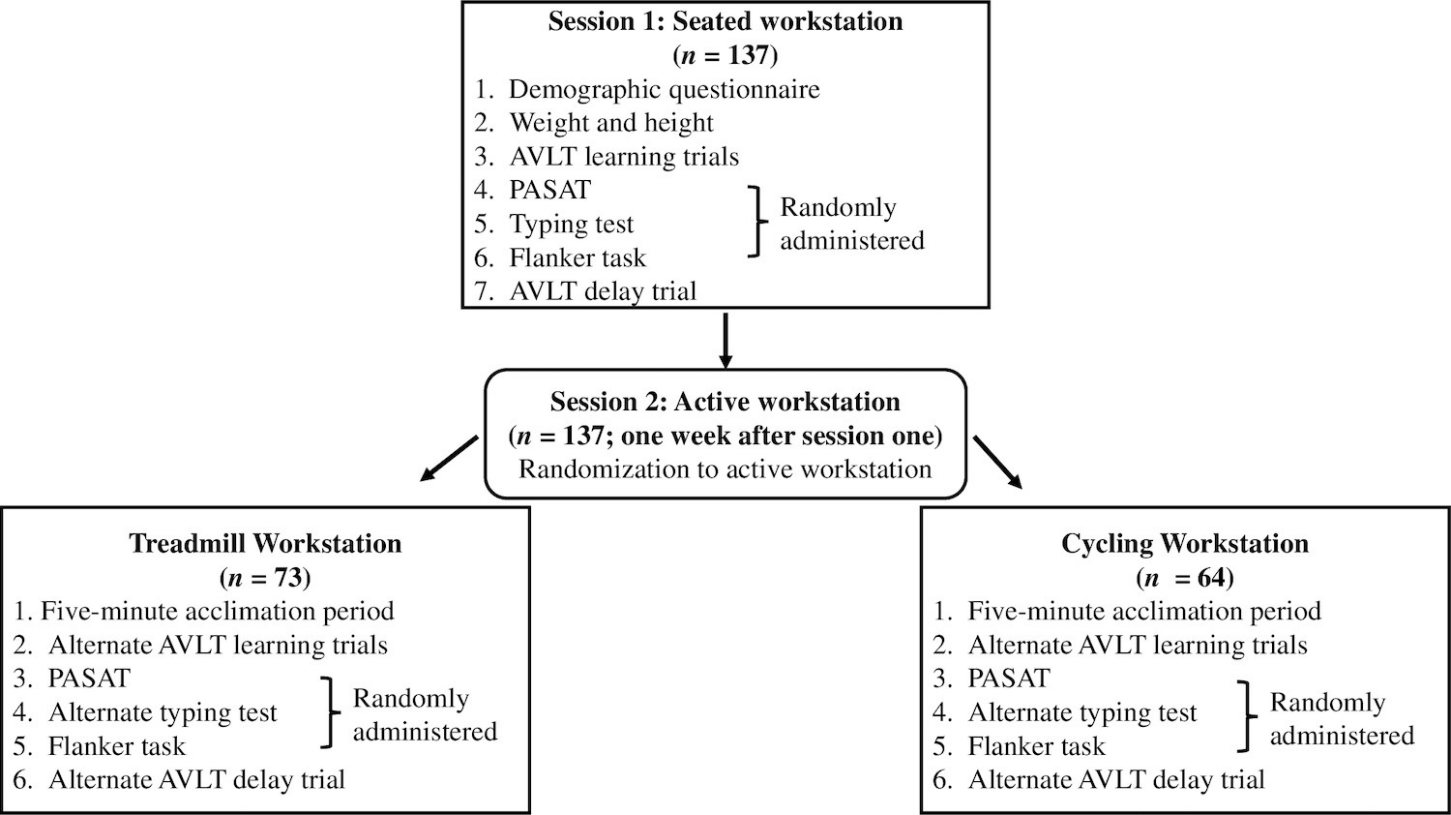
Timeline and description of procedures. AVLT, Rey Auditory Verbal Learning Test; PASAT, Paced Auditory Serial Addition Test.

We used a mixed research design, with baseline to post-testing being a within-subjects factor and active workstation group (treadmill versus cycling desk) being a between-subjects factor (see Fig 1 for visualization of study procedures). Specifically, participants attended two days of testing: a seated session and an active workstation (either treadmill desk or cycling desk) session seven days after the seated session [22, 26]. When participants arrived at both sessions, research assistants verbally confirmed that participants had not consumed caffeine or participated in vigorous exercise in the previous 24 hours, had not consumed food in the last four hours, and had obtained at least 7 hours of sleep the previous night in order to control for potential effects of exercise, caffeine, food, and sleep on cognition [28].

At the first session all participants provided demographic information and weight and height were obtained using an eye level beam physician scale with height rod (Detecto, Webb City, MO). All participants performed four tests seated in a comfortable position at a table during the first session in order to establish a baseline score for each test: Rey Auditory Verbal Learning Test (AVLT), Paced Auditory Serial Addition Test (PASAT), a typing test, and a flanker task [described below] [29–31]. Participants performed the AVLT first to allow for adequate time between learning and delay tasks (30 minutes). The other three tasks (PASAT, typing task, flanker task) were randomly administered after the AVLT learning trials. Participants then performed the delayed recall portion of the AVLT.

For the second session, participants were randomized for their active workstation session to either the treadmill group or the cycling group. At the beginning of the second session, participants were given five minutes of self-paced walking or cycling to acclimate to the treadmill or cycling desk prior to starting the cognitive tasks. Participants assigned to the treadmill group slowly walked (1.5 mph (2.4 km/h), 0% grade) at a treadmill (Pro-Form, Logan, UT) with a desk (TrekDesk, Oklahoma City, OK) placed over the treadmill. Desk dimensions were 183 cm x 86 cm. Treadmill desks were adjusted to a height that the participant confirmed comfortable for typing. Participants assigned to the cycling group slowly pedaled maintaining a rate of 7 mph (11.3 km/h) with no active resistance on a FitDesk 2.0 desk exercise bike (Kernersville, NC). Bike dimensions were 71 cm x 41 cm x 114 cm. The seat was adjusted to a height the participant confirmed comfortable for pedaling. As the participants cycled/walked, they performed the same tests that were performed during the first session with randomized order (with the exception of AVLT). Alternate test forms were used for the AVLT and typing test.

### Measures

#### Paced auditory serial addition test

In the PASAT, participants were asked to sum two numbers between 1 and 9 presented to them in an audio recording for four separate trials of 25 items each. The amount of time between item presentation was consistent within trial, but decreased between trials as trials progressed from one to four: 2.4 s, 2.0 s, 1.6 s, and 1.2 s, respectively. The duration of actual number presentation was 0.4 s per number. The PASAT is a measure of attention, as supported by a factor analytic study in which the PASAT loaded on a freedom from distractibility factor derived from a Wechsler intelligence test at .46 [32]. More specifically, in another study the PASAT measured speed of processing, working memory and sustained memory [33]. The PASAT in other samples had a correlation between items from .76-.95 and test-retest correlations ranging from .9-.97 depending on the study and sample [33]. Of note, the PASAT was sensitive to practice effects at several intervals [33]. We used the number correct (both summed across all four trials and per each trial) as the outcome variable instead of accuracy as some participants strategically omit items to do better with the timed task [22, 34].

#### Flanker task

In a flanker task, participants were instructed to respond as quickly and accurately as possible to indicate the direction of a target arrow (middle arrow) in congruent (e.g., <<<<<) and incongruent (e.g., <<><<) trials using their index finger for a left arrow and middle finger for a right arrow. All stimuli were presented in white text on a black background, approximately 20” from the participant’s head on a computer screen: the fixation cross appeared for 500 ms; the four flanker arrows appeared for 110 ms; the target arrow appeared 80 ms after onset of flanker arrows and remained on the screen for 30 ms. After participants responded, a black screen appeared for 500 ms, followed by a feedback screen for 1,000 ms, indicating whether or not the participant responded correctly. Forty-five percent of trials were congruent and 55% were incongruent. We measured the reaction time and accuracy by congruency for each of the participants.

A flanker task is often considered a measure of attention and cognitive control [the ability to organize and plan ahead to complete tasks] [35]. Cognitive monitoring and attentional processes in a flanker task has been validated through N2 and P3 event-related potential (ERP) components in EEG research [35]. Moreover, in a large battery of attention tests, a flanker task loaded on an executive control aspect of attention [36]. In a small sample of 22, the Cronbach’s alpha for mean reaction time varied from .84-.94 depending on trial type [37]. In the same sample, test-retest reliability at 11 weeks varied from .73-.82 depending on trial type [37]. We used the difference in mean reaction time between congruent and incongruent trials on correct trials and accuracy for incongruent trials as outcome variables. The method in the current paper indicates more specific parameters for flanker outcomes (“mean response time on correct trials”) than in the pre-registered trial.

#### Rey auditory verbal learning test

The AVLT is a measure of list-learning memory that consists of word recall from two 15-item word lists. Participants were asked to recall words for each trial after hearing the first list in five consecutive trials. Next, participants were asked to recall words from an interference list of 15 words for one trial. Participants then immediately recalled the original list of words. After a 30-minute delay, participants again recalled the original list of words.

The AVLT is a measure of verbal memory, as validated in one sample in which AVLT scores were significantly lower in the group with impaired memory as measured by the Wechsler Memory Scale (WMS) compared to a group with typical memory as measured by the WMS [38]. Test-retest reliability ranged from .61-.86 for trial scores and .51-.72 for delayed recognition and recall when tested one month apart; reliability for trial scores was lower and reliability for delayed recall scores was higher in another sample tested two weeks apart [39]. We used the sum of number correct on trials 1–5, individual trial scores, short delay recall, and the 30-minute delay recall as outcome variables in order to assess learning and memory.

#### Typing test

Participants were asked to type words presented on a screen for 10 minutes as quickly as they could without making errors using TypingMaster Pro, version 6.3. Participants were able to self-correct mistyped words while typing within the word; however, after typing the full word followed by a space, the mistype was considered an error and participants could not self-correct. We did not find any literature describing the reliability and validity of this task. We assessed gross typing speed in words typed per minute, net typing speed in words typed per minute minus errors (gross speed subtracting errors), and accuracy (net typing speed/gross typing speed). We used net typing speed, gross typing speed, and accuracy as the outcome variables in our study.

### Data analysis

We used Stata version 16 to clean data and SPSS version 25 for all subsequent data analyses (see https://osf.io/4qz3k/ for raw data and scripts). We corrected for multiple comparisons in our use of analysis of variance (ANOVA) tests by *a priori* setting the *p* value alpha cut off to less than or equal to .01 for statistical significance for all tests. We decomposed any .01 or lower significant interaction or main effect with tests of simple effects. We additionally corrected for multiple comparisons (Bonferroni) in follow-up tests for main effects which included more than two levels (e.g., AVLT trials 1–4, PASAT trials 1–4). For ANOVA models, we used Greenhouse-Geisser corrections when there were more than two levels of a factor to correct for possible violations of sphericity. Partial-η^2^ is reported as a measure of effect size.

#### Data screening

As indicated in our pre-registration, we first assessed the raw data for missing values, normality, and outliers. Thirty-six data points were three or more interquartile range beyond the median and were fenced within the third interquartile range. Approximately 1.2% of our total data was missing; nine participants were missing flanker data and two participants were missing typing data due to computer malfunction. None of the missing data were significantly correlated with other variables suggesting the missingness was random; thus, we chose to leave the missing data in its original form. We examined possible transformations for any non-normal variables. AVLT trials, PASAT trials, flanker tasks, PASAT summed score, and typing accuracy scores were non-normal as indicated in Stata version 16, with the *mvtest* command. We transformed the PASAT summed score and flanker reaction time scores by squaring the PASAT data and taking the square root of the flanker data. For all other non-normal data, transformations did not significantly improve normality, thus we kept them in their original format.

#### Confirmatory factor analysis

We planned to perform confirmatory factor analysis (CFA) *a priori*, with subsequent plans to run a second model (see pre-registration at https://osf.io/f4ej3) should the first model not converge. None of the CFA models converged (see https://osf.io/4qz3k/ for more detail on CFA models). Thus, in order to answer our questions and hypotheses, we performed separate repeated measures ANOVA models, as proposed in our *a priori* statistical method (see https://osf.io/f4ej3). No further information is presented in the current manuscript given the models did not converge.

#### Mixed-design analysis of variance

In order to answer our research questions and replicate a similar study by Larson, LeCheminant [22], we performed a total of eleven separate mixed-design ANOVA models. Seven of the eleven models were 2-Group x 2-Session models with each respective outcome as the dependent variable: PASAT summed score, AVLT trials 1–5 summed score, AVLT 30-minute delay recall score, flanker incongruent accuracy score (flanker score was mis-entered in the pre-registration as congruent instead of incongruent), gross typing speed, net typing speed, and typing speed accuracy. The remaining four of the eleven mixed-design ANOVA models were 2-Group x 2-Session x Trial (number varied depending on outcome) models with the following dependent variables: reaction time to correct flanker trials (2 trial types: congruent and incongruent), AVLT score for learning trials (5 trial types: trials 1–5), AVLT score for recall trials (2 trial types: short delay and 30-minute delay), PASAT score for each trial (4 trial types: trials 1–4).

## Results

### Hypothesis one: Is there a difference in cognitive performance between seated (session one) and active (session two) workstations?

**Fig 2:**
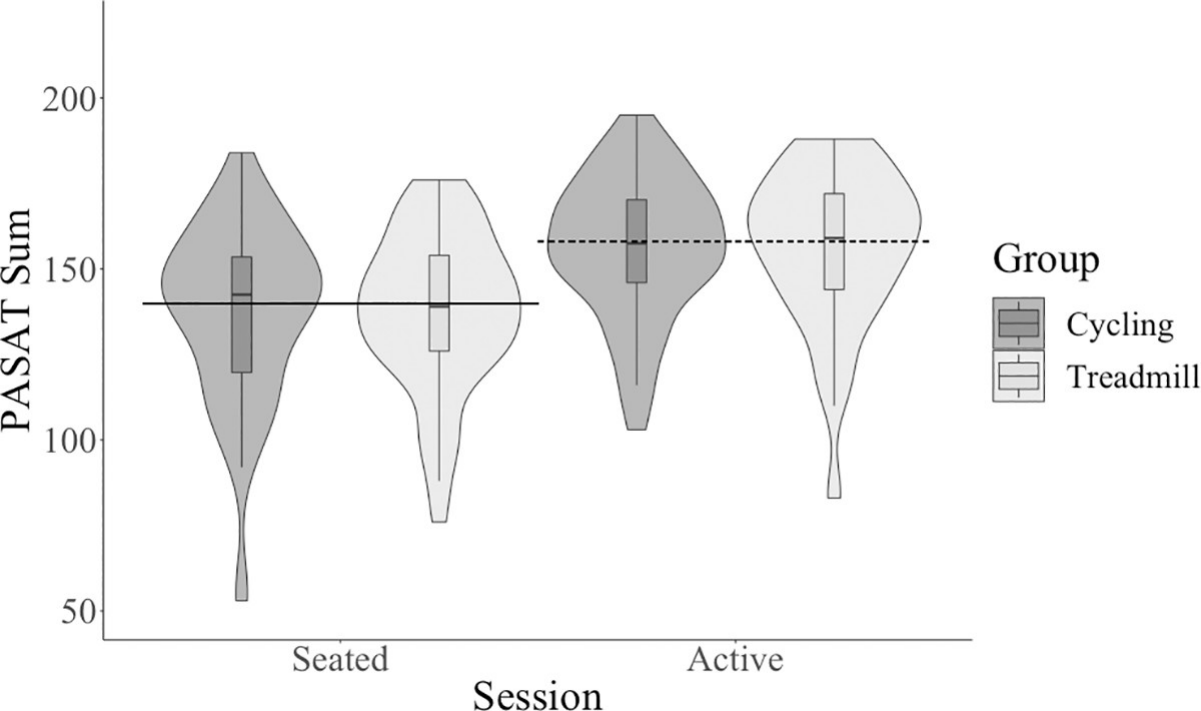
PASAT summed score across session and between type of active workstation. PASAT, Paced Auditory Serial Addition Test.

For mean comparisons between groups and sessions for all variables, see S1 Table. Participants showed increased performance on the PASAT summed score during the active condition compared to the seated condition; the main effect of session was significant with a large effect size, see Table 1 and Fig 2.

**Table 1:** Main effects of session (seated workstation or active workstation) on cognitive and typing performance.

Outcome	*df*	*F*	η_*p*_^2^	*p*
**AVLT: sum**	1, 135	10.49	.07	.002
**AVLT: 30-minute delay**	1, 135	39.31	.23	< .001
**AVLT: short and 30-minute delay**	1, 135	29.87	.18	< .001
**PASAT: sum**	1, 135	301.65	.69	< .001
**Typing: net**	1, 133	16.58	.11	< .001
**Typing: gross**	1, 133	13.36	.09	< .001
**Typing: acc.**	1, 133	6.95	.05	.009
**Flanker: acc.**	1, 126	7.71	.06	.006
**Flanker RT**	1, 126	71.26	.36	< .001
**Flanker RT: cong vs. incong**^**a**^	1, 126	909.11	.88	< .001
**Flanker RT (session x trial)**^**b**^	1, 126	9.80	.07	.002

AVLT, Rey Auditory Verbal Learning Test; PASAT, Paced Auditory Serial Addition Test; RT, reaction time.

^**a**^collapsed across session.

^b^interaction term.

**Fig 3:**
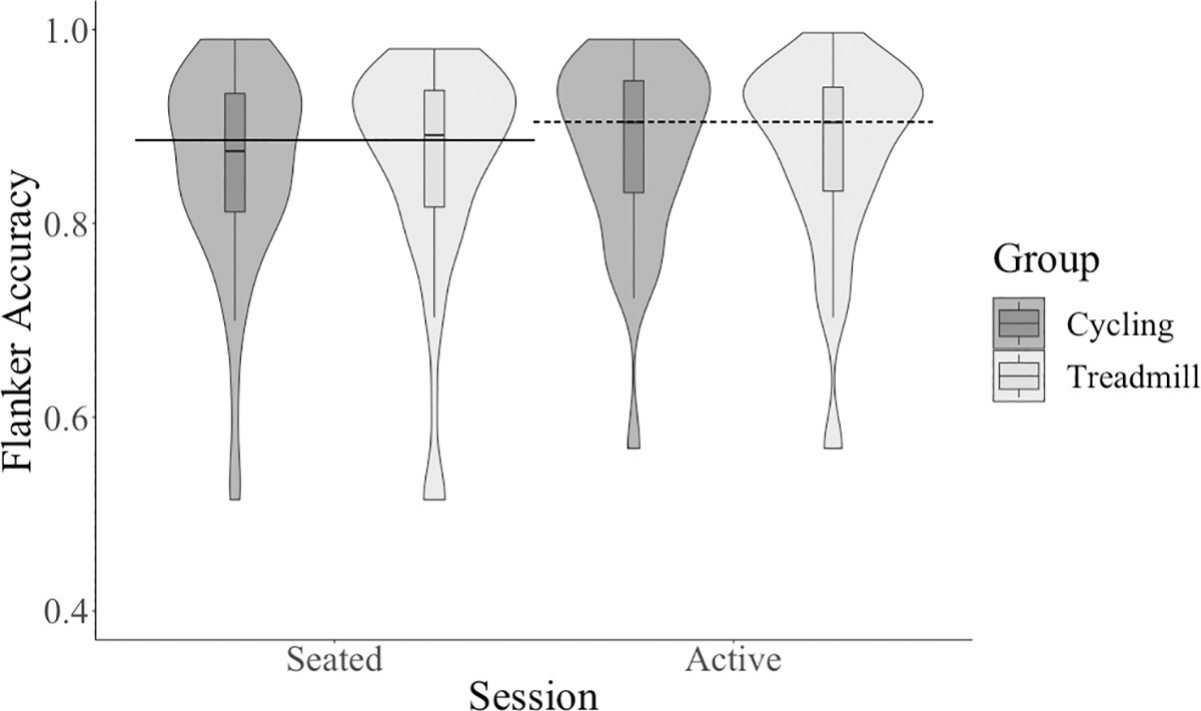
Accuracy for incongruent flanker trials across session and between type of active workstation.

Participants had increased performance on accuracy scores for flanker incongruent trials at the active condition compared to the seated condition with a small effect size, see Table 1 and Fig 3. On correct trials, participants performed faster on flanker tasks at the active condition than the seated condition with a large effect size, see Table 1. Collapsed across session, participants performed faster on congruent trials than on incongruent trials with a large effect size, see Table 1. There was a significant session by trial interaction for flanker reaction times with a small effect size, see Table 1, such that the difference in reaction time between congruent and incongruent trials at the active condition was smaller than the difference in reaction time between congruent and incongruent trials at the seated condition, *t*(127) = 3.10, *p* = .002.

**Fig 4:**
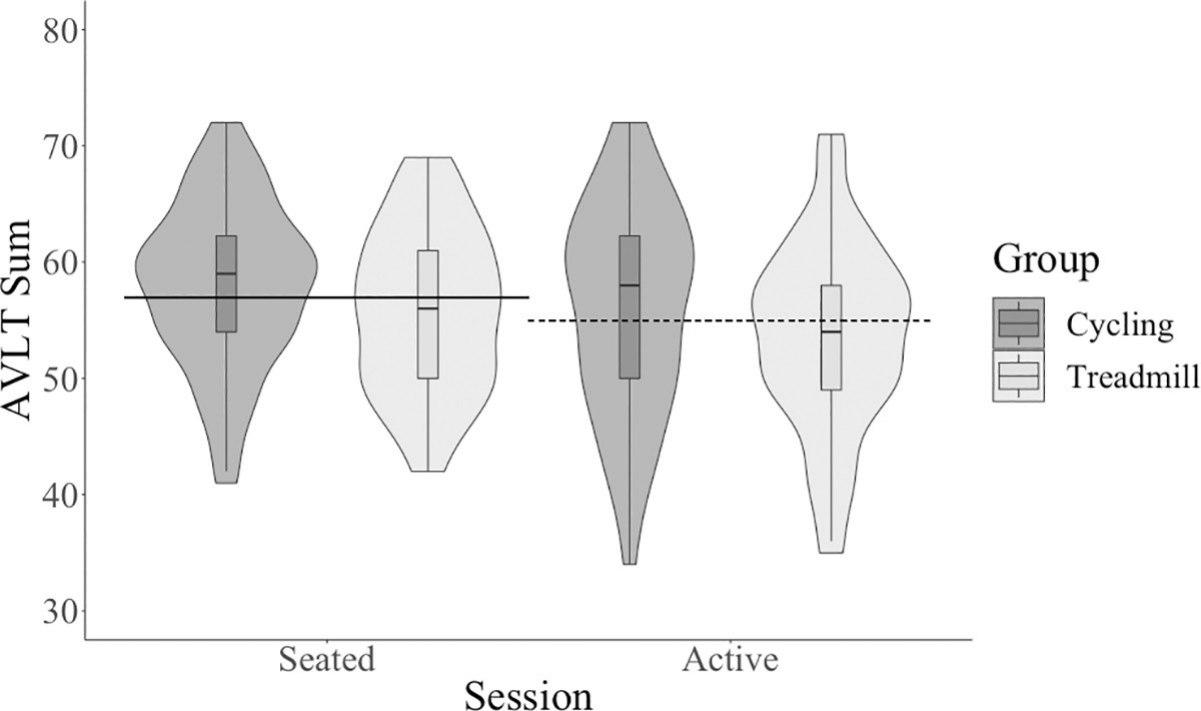
AVLT summed score across session and between type of active workstation. AVLT, Rey Auditory Verbal Learning Test.

**Fig 5:**
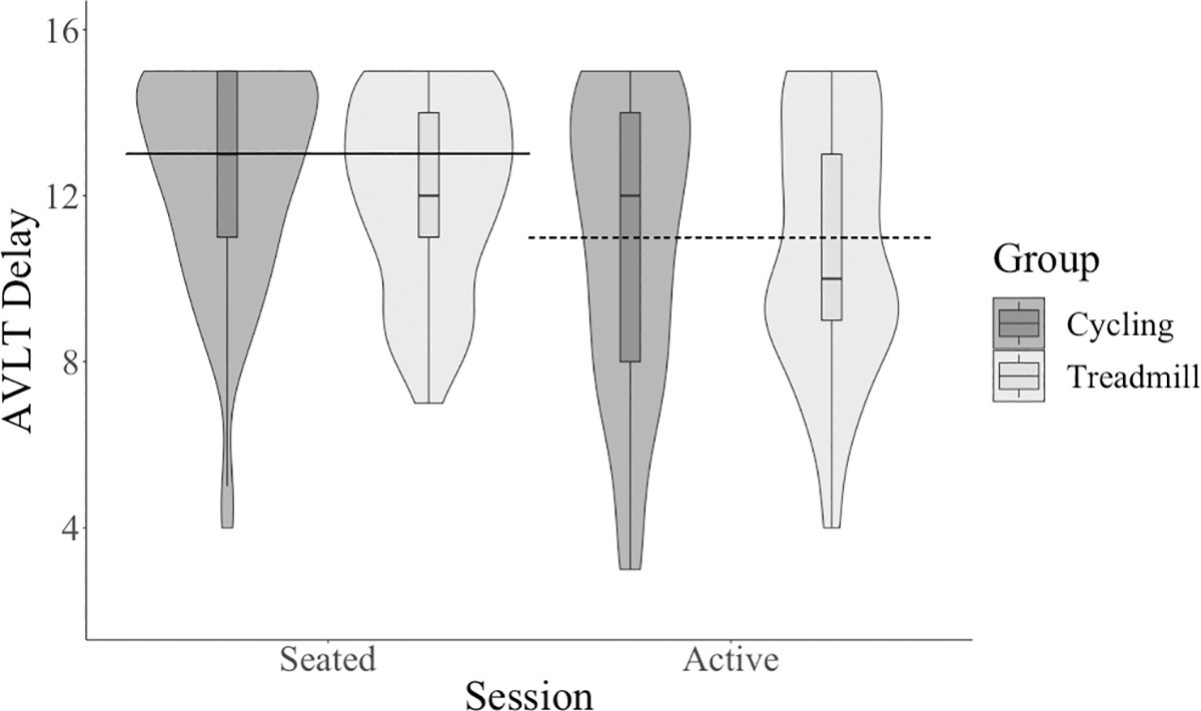
AVLT 30-minute delay score across session and between type of active workstation. AVLT, Rey Auditory Verbal Learning Test.

Participants had decreased performance on the AVLT summed score at the active condition compared to the seated condition with a small effect size, see Table 1 and Fig 4. Participants had decreased performance on the 30-minute delay AVLT trial at the active condition compared to the seated condition with a medium effect size, see Table 1 and Fig 5. In summary, contrary to our hypothesis, there was a difference between the seated condition and the active condition on all cognitive scores with varied effect sizes: performance on cognitive tests was better for PASAT and flanker tests but decreased for AVLT trials at the active condition compared to the seated condition.

### Hypothesis two: Is there a difference in typing skills between seated (session one) and active (session two) workstations?

**Fig 6:**
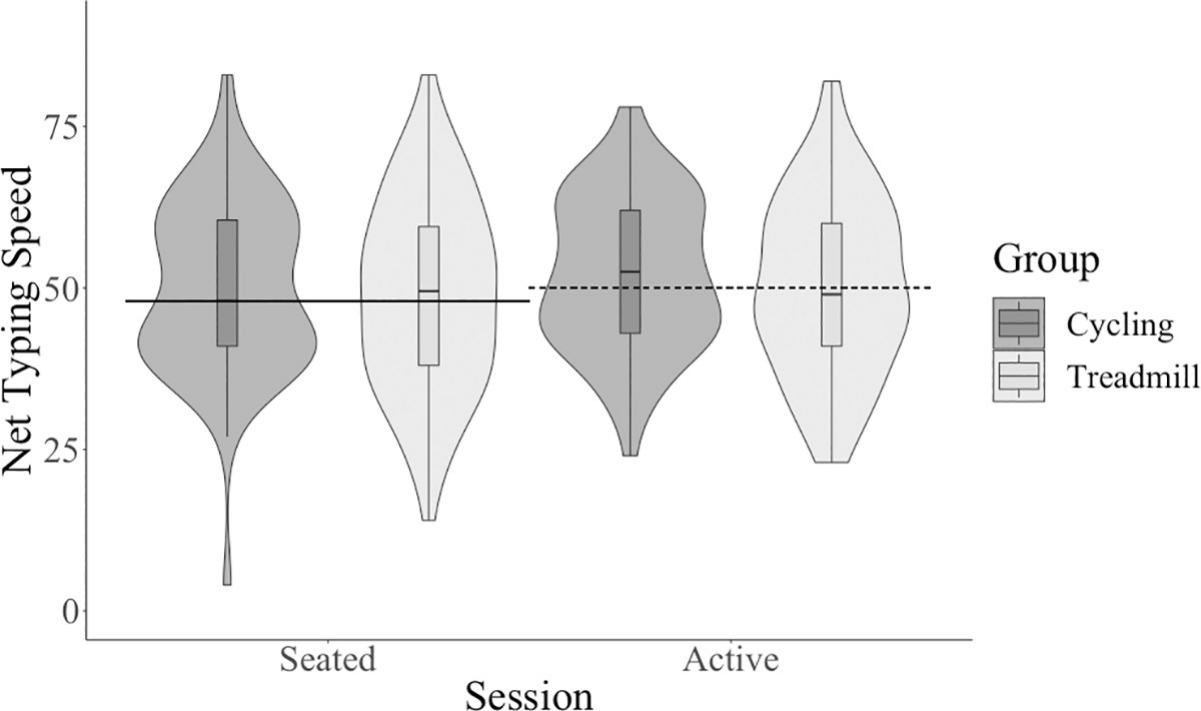
Net typing speed across session and between type of active workstation.

**Fig 7:**
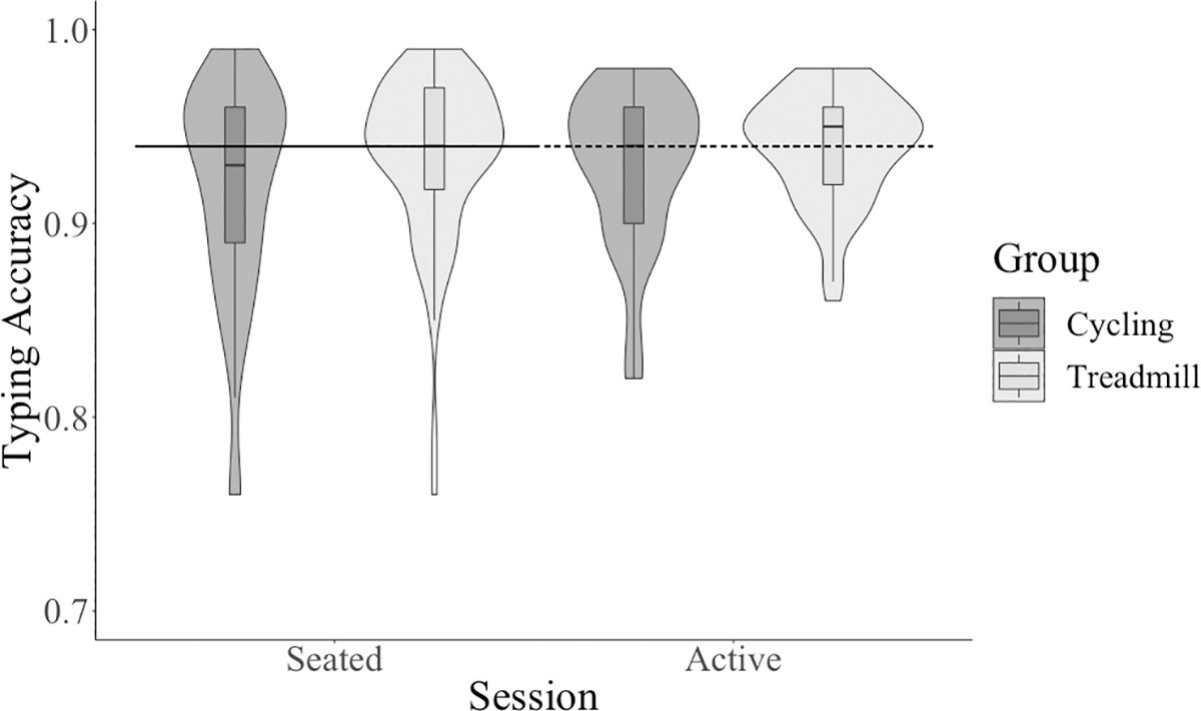
Typing accuracy across session and between type of active workstation.

Participants performed better on gross typing speed at the active condition compared to the seated condition with a medium effect size, see Table 1. Participants performed better on net typing speed at the active condition compared to the seated condition, with a medium effect size, see Table 1 and Fig 6. Participants performed with increased typing accuracy at the active condition compared to the seated condition, with a small effect size, see Table 1 and Fig 7. Overall, contrary to our hypothesis, participants typed faster and more accurately during the active condition compared to the seated condition with a medium and small effect size, respectively.

### Hypothesis three: Is there a difference in cognitive performance between cycling and treadmill?

There was no significant (*p* < .01) main effect of group (cycling and treadmill) on any cognitive outcome (see Table 2): PASAT sum (see Fig 2), AVLT sum (see Fig 4), AVLT 30-minute delay trial (see Fig 5), flanker accuracy on incongruent trials (see Fig 3), flanker reaction time. There were also no significant interactions between session and group for any cognitive outcome (see Table 3): PASAT sum, AVLT sum, AVLT 30-minute delay trial, flanker accuracy on incongruent trials, flanker reaction time.

**Table 2:** Main effects of group (cycling or treadmill) on cognitive and typing performance.

Outcome	*df*	*F*	η_*p*_^2^	*p*
**AVLT: sum**	1, 135	3.97	.03	.048
**AVLT: 30-minute delay**	1, 135	0.32	.00	.571
**PASAT: sum**	1, 135	0.00	.00	.990
**Typing: net**	1, 133	0.48	.00	.488
**Typing: gross**	1, 133	1.76	.01	.187
**Typing: acc.**	1, 133	4.17	.03	.043
**Flanker: acc.**	1, 126	0.01	.00	.916
**Flanker: RT**	1, 126	0.01	.00	.933

AVLT, Rey Auditory Verbal Learning Test; PASAT, Paced Auditory Serial Addition Test; RT, reaction time.

**Table 3:** Session by group interactions on cognitive and typing performance.

Outcome	*df*	*F*	η_*p*_^2^	*p*
**AVLT: sum**	1, 135	0.02	.00	.904
**AVLT: 30-minute delay**	1, 135	0.49	.00	.484
**PASAT: sum**	1, 135	0.00	.00	.948
**Typing: net**	1, 133	1.08	.01	.300
**Typing: gross**	1, 133	0.06	.00	.808
**Typing: acc.**	1, 133	1.43	.01	.235
**Flanker: acc.**	1, 126	0.13	.00	.724
**Flanker: RT**	1, 126	0.04	.00	.845

AVLT, Rey Auditory Verbal Learning Test; PASAT, Paced Auditory Serial Addition Test; RT, reaction time.

### Hypothesis four: Is there a difference in typing skills between cycling and treadmill?

There was no significant main effect of group (cycling and treadmill) on any typing outcome (see Table 2): gross typing speed, net typing speed (see Fig 6), typing accuracy (see Fig 7). There were no significant interactions between session and group for any typing outcome (see Table 3): gross typing speed, net typing speed, typing accuracy.

### Additional ANOVAS for replication: Trials on the PASAT and AVLT tasks

There was a main effect of trial on PASAT score with a large effect size, see Table 4. Participants had decreased PASAT performance as trials increased (e.g., speed of presentation increased), all follow-up comparisons between trials were significant at a *p* < .001 level. There was a significant interaction between session and trial on PASAT score for trials 1–4 with a small effect size, see Table 4. Performance on all PASAT trials was increased at the active condition compared to the seated condition (all follow-up comparisons significant at a *p* < .001 level).

**Table 4:** Main effects and interactions of trials on the PASAT and AVLT.

Outcome	*df*	*F*	η_*p*_^2^	*p*
**Main Effects of Trial**				
**AVLT**	3.22, 435.07	946.87	.88	< .001
**AVLT: short and 30-minute delay**	1, 135	26.37	.16	< .001
**PASAT**	2.46, 332.34	789.67	.85	< .001
**Session by Trial Interaction Terms**				
**AVLT**	3.48, 469.38	7.60	.05	< .001
**AVLT: short and 30-minute delay**	1, 135	16.99	.11	< .001
**PASAT**	2.96, 398.90	4.22	.03	.006
**Group by Trial Interaction Terms**				
**AVLT**	3.22, 435.07	0.50	.00	.696
**AVLT: short and 30-minute delay**	1, 135	0.91	.01	.343
**PASAT**	2.46, 332.34	0.29	.00	.792

AVLT, Rey Auditory Verbal Learning Test; PASAT, Paced Auditory Serial Addition Test; RT, reaction time.

There was a main effect of trial on AVLT scores on trials 1–5 with a large effect size, see Table 4. Participants had increasing AVLT performance as trials increased, all follow-up comparisons between trials were significant at a *p* < .001 level. There was a significant interaction between session and trial on AVLT score for trials 1–5 with a small effect size, see Table 4. That is, later AVLT trial (trials 3,4, and 5) performance was significantly lower at the active condition compared to the seated condition, *t*(136) = 4.70, *p* < .001; *t*(136) = 3.78, *p* < .001; *t*(136) = 3.70, *p* < .001, respectively, but earlier AVLT trial (trials 1 and 2) performance was not significantly different between active and seated conditions, *t*(136) = -0.97, *p* = .334; *t*(136) = 1.22, *p* = .224), respectively.

There was a main effect of session on AVLT delay score with a medium effect size: participants had decreased performance on delay trials at the active condition compared to the seated condition, see Table 1. There was a main effect of trial on AVLT delay scores with a medium effect size: participants had decreased performance on the 30-minute delay trial compared to the short delay trial, see Table 4. There was a significant session by trial interaction on AVLT delay score, with a medium effect size, see Table 4. At the active condition, participants had significantly decreased scores on the 30-minute delay trial compared to the short delay trial, *t*(136) = 6.31, *p* < .001, but no significant difference between delay trial performance at the seated condition, *t*(136) = 1.22, *p* = .225.

There were no significant interactions between group and trial, see Table 4. Overall, there were significant main effects of session and trial on PASAT and AVLT scores (for both learning and delay trials), many of them large effects. There was also an interaction of session and trial on PASAT and AVLT scores (for both learning and delay trials), with small to medium effect sizes.

## Discussion

We tested the difference in cognitive outcomes between seated and active workstation conditions. We found significant differences between the seated condition and the active workstation condition: PASAT scores, flanker accuracy, and flanker speed were higher/faster and AVLT scores (learning trials and delay) were lower during the active workstation condition compared to the seated condition. Next, we tested the difference in typing outcomes between a seated condition and an active workstation condition. Typing speed (net and gross) and accuracy were increased during the active workstation condition compared to the seated condition. Finally, we tested the difference in cognitive and typing outcomes between types of active workstations (treadmill desk or cycling desk). We did not find significant differences on any cognitive or typing performance between type of workstation (treadmill or cycling).

The improved scores on tasks assessing attention and cognitive control during the active workstation compared to the seated condition is somewhat surprising given most studies find cognitive skills such as attention, working memory, processing speed, inhibition, and cognitive control are not significantly different between control and active workstations [5, 6, 11, 13, 15, 40, 41]. The faster typing speed at active workstations compared to sitting workstations is also unlike the majority of studies which have found no significant effect or detrimental effect of active workstation on typing performance [5, 6, 15, 19, 20, 22–25, 27, 40–43].

However, a few studies suggest improvement in cognitive performance such as attention, working memory, and executive functioning at active workstations compared to seated workstations [20, 21, 44]. The improvement seen in attention, cognitive control, and typing speed in the current study could be attributed to the generally faster reaction time across cognitive tasks [20, 44, 45]. The mechanism by which exercise may specifically reduce reaction time is unclear. Some authors argue for the facilitative effects of submaximal aerobic exercise on information processing [45]. Tomporowski [45] and Arcelin, Delignieres [46] suggest faster reaction time during exercise may be explained by preparing participants to respond to incoming stimuli, speeding up information processing. However, other authors suggest improvement at later stages in the response [motor stage; [20]. Perhaps more efficient peripheral motor functioning during exercise, such as changes in electromechanical transduction within the muscle fibers, explain the reduced reaction time and thereby improved performance on cognitive tasks [20, 44]. Another possible mechanism is simple practice effects. Participants were more familiar with the tasks from the seated session to the active session. Even if practice effects are the primary mechanism, this is still meaningful as it suggests the active workstations are not detrimental to reaction times or cognitive performance in ways that would strongly interfere with simple cognitive performance.

Differences in findings between the current study and other studies may be due to a variety of factors. First, previous active workstation studies often utilized small sample sizes (many less than 50 participants) and may have had insufficient power to detect a true effect [15, 23–25, 27, 40, 42, 43]. Second, theories of optimal exercise arousal on cognitive performance [45] and the moderating effect of workstation intensity on outcome [23, 24] suggest speed/intensity at active workstation likely moderates outcomes. Specifically, some studies suggest the relationship between exercise and cognition follows an inverted U-shaped function in which moderate levels of exercise (as opposed to resting state or intense levels of exercise) are associated with the best cognitive performance, especially for reaction time tasks [45]. Perhaps the intensities utilized in the current study are optimal for cognitive and typing outcomes. Finally, unlike the majority of previous studies, the current study did not randomize or counter-balance the seated session and active session. Thus, improvements seen in cognitive and typing tasks at the active session may also be explained by practice effects as noted above.

Interactions between session and trial for both AVLT learning and delay trials suggest active workstations compared to seated workstations have a somewhat detrimental effect specifically on later learning trials and delay trials, perhaps suggestive of a targeted detrimental effect on retrieval/long-term memory as opposed to attention/encoding. Little work has been done on long-term memory performance during active workstations: one study suggests detrimental effects of active workstations on learning scores [22], although other evidence suggests no significant difference on learning/memory measures between seated and active workstations [20]. Increased forgetting at the active workstation may be explained by the increased non-specific retroactive interference induced by dual-task performance [e.g., walking, cycling] [47, 48]. The specific differential effects of active workstation on cognitive task as seen in the current study (cognitive control and attention versus learning/memory tasks) may be explained by the benefits of exercise to information processing and processing speed as explained above, thus improving performance on timed versus untimed tasks [26, 44, 45, 49].

The results of the current study suggest no significant differences in outcome between types of workstations (treadmill versus cycling), whether measuring cognitive or typing performance. The nonsignificant differences in cognitive performance between types of active workstations are expected and corroborated by other research [21, 27]. However, the nonsignificant difference in *typing* outcomes between type of active workstation are unexpected: research to this point suggests a relatively decreased typing performance at a treadmill desk compared to a cycling desk [23, 27]. Because the majority of previous studies do not directly compare the effects of different active workstations, it is possible the inferred differences between types of active workstations on cognitive and typing outcomes are inaccurate.

The current study significantly contributes to the current work on active workstations through several strengths: randomly assigned active workstation conditions, direct comparison between active workstations, relatively large sample size, appropriate measures with adequate internal and test-retest reliability and adequate construct validity, and a familiarization period for active workstations to reduce confounds of apprehension and distraction with a new task. Some limitations may have also influenced the results of the current study. For example, because the seated and active workstation conditions were not counter-balanced (active conditions always happened after the seated baseline) the results were subject to practice effects, perhaps leading to artificially improved typing and cognitive performance at the active workstation condition compared to the seated condition. However, the use of available alternate versions for tests (AVLT, typing task; or random trials at sessions for flanker) and typically high test-retest reliability for measures likely ameliorated practice effects to some extent. The convenience sample used in the current study (undergraduate students) limits the generalizability of the current results to other settings, such as typical workplace environments. Additionally, participants in the current study constitute a healthy, young population that is affected at a much lower rate by the health concerns that active workstations are designed to help prevent; namely, diabetes, obesity, and cardiovascular disease. Finally, the short timeline used in the current study (two one-hour sessions) is not easily generalizable to the workplace in which an active workstation may be used (e.g., several hours per day across months or years).

The lack of decreased performance in areas such as cognitive control, attention, and typing between seated conditions and an active workstation in the current study supports a consideration of active workstation implementation for not only the workplace but environments that would benefit from breaks in sedentary behavior, including classrooms [50]. Given the documented health benefits of walking or cycling during work, the absence of considerable detriments to cognitive performance would seem to suggest that the use of alternative workstations is beneficial to traditionally sedentary environments [51]. One factor not investigated in this study is the emotional response of individuals working at active workstations. Some studies have suggested one significant advantage of active workstation use is the increased motivation and positive mood individuals experience when working, which might extend to more daily physical activity [52, 53]. Future studies may consider investigating emotional responses to active workstations, the impact of long-term active workstation use on cognitive and typing performance, and the impact of active workstation on complex work activities such as writing and problem solving.

## Supporting information

S1 Table Cognitive outcomes at seated, treadmill, and cycling condition. (PDF)
