# Supplementary material for: Does type of active workstation matter? A randomized comparison of cognitive and typing performance between rest, cycling, and treadmill active workstations
Source: PLoS One. 2020 Aug 7;15(8):e0237348. doi: 10.1371/journal.pone.0237348 (PMC7413476; doi:10.1371/journal.pone.0237348)
Supplement: S1 Table — (PDF) [file pone.0237348.s001.pdf]

S1 Table. Cognitive Outcomes at Seated, Treadmill, and Cycling Condition.

Outcome	Seated all	Active all	Seated, cycling	Seated, treadmill	Active, cycling	Active, treadmill
	(*n* = 137)	(*n* = 137)	(*n* = 64)	(*n* = 73)	(*n* = 64)	(*n* = 73)
	Mean (*SD*)	Mean (*SD*)	Mean (*SD*)	Mean (*SD*)	Mean (*SD*)	Mean (*SD*)
**AVLT: sum**	56.85 (7.06)	54.72 (8.58)	58.05 (7.02)	55.81 (6.97)	56.00 (8.98)	53.60 (8.11)
**AVLT: 1**	7.36 (1.78)	7.55 (2.00)	7.72 (1.88)	7.05 (1.63)	7.77 (2.13)	7.37 (1.87)
**AVLT: 2**	10.39 (2.03)	10.13 (2.29)	10.67 (2.08)	10.15 (1.98)	10.50 (2.34)	9.81 (2.21)
**AVLT: 3**	12.34 (1.86)	11.47 (2.15)	12.44 (1.99)	12.26 (1.75)	11.75 (2.17)	11.23 (2.12)
**AVLT: 4**	13.13 (1.71)	12.49 (2.05)	13.38 (1.54)	12.93 (1.84)	12.75 (2.11)	12.26 (1.98)
**AVLT: 5**	13.61 (1.43)	13.07 (1.77)	13.84 (1.43)	13.41 (1.41)	13.23 (1.84)	12.93 (1.71)
**AVLT: imm.**	12.41 (2.35)	11.68 (2.58)	12.72 (2.46)	12.15 (2.23)	11.82 (2.61)	11.55 (2.57)
**AVLT: delay**	12.27 (2.40)	10.86 (3.19)	12.48 (2.43)	12.08 (2.27)	10.91 (3.44)	10.82 (2.98)
**PASAT: sum**	137.55 (24.20)	156.31 (21.74)	137.28 (25.90)	137.79 (22.78)	156.41 (20.66)	156.22 (22.78)
**PASAT: 1**	42.24 (6.38)	45.89 (3.72)	42.08 (6.27)	42.40 (6.52)	45.66 (3.81)	46.10 (3.64)
**PASAT: 2**	36.37 (7.32)	41.60 (6.16)	36.25 (8.08)	36.48 (6.65)	41.61 (6.32)	41.59 (6.07)
**PASAT: 3**	32.39 (6.99)	37.70 (6.68)	32.39 (7.41)	32.40 (6.64)	37.92 (5.97)	37.51 (7.28)
**PASAT: 4**	26.48 (6.65)	31.27 (7.09)	26.44 (7.12)	26.52 (6.27)	31.34 (6.87)	31.21 (7.32)
**Typing: net**	49.47 (14.16)	51.23 (12.80)	50.05 (13.41)	48.96 (14.86)	52.28 (11.81)	50.32 (13.63)
**Typing: gross**	53.31 (13.30)	54.45 (12.93)	54.84 (12.33)	51.97 (14.05)	56.00 (11.73)	53.08 (13.83)
**Typing: acc.**	.93 (.05)	.93 (.03)	.92 (.06)	.94 (.04)	.93 (.04)	.94 (.03)
**Flanker: acc.**	.86 (.11)	.88 (.10)	.86 (.10)	.86 (.12)	.88 (.09)	.87 (.10)

**Flanker RT: cong. (ms)**	415.28 (40.07)	398.62 (41.03)	415.28 (40.39)	415.27 (40.08)	399.48 (40.65)	397.83 (41.65)
**Flanker RT: incong. (ms)**	469.35 (46.32)	448.46 (50.11)	468.36 (50.99)	470.26 (41.99)	447.24 (51.46)	449.58 (49.20)

AVLT, Rey Auditory Verbal Learning Test; PASAT, Paced Auditory Serial Addition Test; RT, reaction time.
